# Identification of copy number alterations associated with the progression of DCIS to invasive ductal carcinoma

**DOI:** 10.1186/1897-4287-10-S2-A93

**Published:** 2012-04-12

**Authors:** C Johnson, KL Gorringe, ER Thompson, K Opeskin, SE Boyle, Y Wang, P Hill, GB Mann, IG Campbell

**Affiliations:** 1 VBCRC Cancer Genetics Laboratory, Peter MacCallum Cancer Centre, East Melbourne, Victoria, Australia; 2 Department of Pathology, University of Melbourne, Melbourne, Victoria, Australia; 3 Department of Anatomical Pathology, St Vincent’s Hospital, Fitzroy, Victoria, Australia; 4 Affymetrix Inc., Santa Clara, USA; 5 The Royal Melbourne and Royal Women’s Hospitals, Parkville, Victoria, Australia

## 

Ductal carcinoma *in situ* (DCIS) is a non-obligate precursor to invasive ductal carcinoma (IDC). Identification of the genetic differences between the two lesions may assist in identifying genes that promote the invasive phenotype. To annotate these alterations we analysed 21 breast tumours containing synchronous areas of DCIS and IDC. Tumour cells were microdissected from FFPE tissue and analysed by 300K Molecular Inversion Probe (MIP) copy number arrays. Matched IDC and DCIS showed highly similar copy number profiles (average of 83% of the genome shared). Four regions of loss (3q, 6q, 8p and 11q) and four regions of gain (5q, 16p, 19q and 20) were recurrently affected in IDC but not in the matching DCIS. *CCND1* and *MYC* showed increased amplitude of gain in IDC. One region of loss (17p11.2) was specific to DCIS. Our data shows that DCIS is an advanced pre-invasive tumour with genetic instability and continues to evolve in parallel with co-existing IDC. In the IDC-specific regions of genomic alteration we have identified novel loci as well as genes with previous links to breast cancer progression.

